# Identification of a functional nuclear translocation sequence in hPPIP5K2

**DOI:** 10.1186/s12860-015-0063-7

**Published:** 2015-06-18

**Authors:** Sheila T. Yong, Hoai-Nghia Nguyen, Jae H. Choi, Carl D. Bortner, Jason Williams, Niyas K. Pulloor, Manoj N. Krishnan, Stephen B. Shears

**Affiliations:** Laboratory of Signal Transduction, National Institute of Environmental Health Sciences, National Institutes of Health, 101 T.W. Alexander Drive, Research Triangle Park, NC 27709 USA; Current address: Thermo Fisher Scientific, LSG/Biosciences Division, 3747 N. Meridian Drive, Rockford, IL 61101 USA; Protein Microcharacterization Core Facility, Mass Spectrometry Group, National Institute of Environmental Health Sciences, National Institutes of Health, 101 T.W. Alexander Drive, Research Triangle Park, NC 27709 USA; Program on Emerging Infectious Diseases, DUKE-NUS Graduate Medical School, 8 College Road, Singapore, 169857 Republic of Singapore

**Keywords:** Cell-signaling, Nuclear translocation, Inositol phosphate kinase, Trafficking

## Abstract

**Background:**

Cells contain several inositol pyrophosphates (PP-InsPs; also known as diphosphoinositol polyphosphates), which play pivotal roles in cellular and organismic homeostasis. It has been proposed that determining mechanisms of compartmentation of the synthesis of a particular PP-InsP is key to understanding how each of them may exert a specific function. Human PPIP5K2 (hPPIP5K2), one of the key enzymes that synthesizes PP-InsPs, contains a putative consensus sequence for a nuclear localization signal (NLS). However, such *in silico* analysis has limited predictive power, and may be complicated by phosphorylation events that can dynamically modulate NLS function. We investigated if this candidate NLS is functional and regulated, using the techniques of cell biology, mutagenesis and mass spectrometry.

**Results:**

Multiple sequence alignments revealed that the metazoan PPIP5K2 family contains a candidate NLS within a strikingly well-conserved 63 amino-acid domain. By analyzing the distribution of hPPIP5K2-GFP in HEK293T cells with the techniques of confocal microscopy and imaging flow cytometry, we found that a distinct pool of hPPIP5K2 is present in the nucleus. Imaging flow cytometry yielded particular insight into the characteristics of the nuclear hPPIP5K2 sub-pool, through a high-throughput, statistically-robust analysis of many hundreds of cells. Mutagenic disruption of the candidate NLS in hPPIP5K2 reduced its degree of nuclear localization. Proximal to the NLS is a Ser residue (S1006) that mass spectrometry data indicate is phosphorylated inside cells. The degree of nuclear localization of hPPIP5K2 was increased when S1006 was rendered non-phosphorylatable by its mutation to Ala. Conversely, a S1006D phosphomimetic mutant of hPPIP5K2 exhibited a lower degree of nuclear localization.

**Conclusions:**

The current study describes for the first time the functional significance of an NLS in the conserved PPIP5K2 family. We have further demonstrated that there is phosphorylation of a Ser residue that is proximal to the NLS of hPPIP5K2. These conclusions draw attention to nuclear compartmentation of PPIP5K2 as being a physiologically relevant and covalently-regulated event. Our study also increases general insight into the consensus sequences of other NLSs, the functions of which might be similarly regulated.

**Electronic supplementary material:**

The online version of this article (doi:10.1186/s12860-015-0063-7) contains supplementary material, which is available to authorized users.

## Background

Inositol pyrophosphates (PP-InsPs; also known as diphosphoinositol polyphosphates) are densely phosphorylated cellular signals that play pivotal roles in cellular and organismic homeostasis [1–4]. The PP-InsPs include 1-PP-InsP_5_ (1-InsP_7_), 5-PP-InsP (5-InsP_7_), and 1,5-(PP)_2_-InsP_4_ (InsP_8_); these signaling molecules are formed by two groups of kinases, PPIP5Ks and IP6Ks. The 5-kinase activities of the three mammalian isoforms of IP6K (Kcs1 in yeasts) convert InsP_6_ and 1-InsP_7_ to 5-InsP_7_ and InsP_8_ [5–8]. PPIP5K1 and PPIP5K2 are 1-kinases that phosphorylate InsP_6_ and 5-InsP_7_ to 1-InsP_7_ and InsP_8_ respectively [8–12]. Homologues of these 1-kinases are expressed throughout metazoans, and also in yeasts (Vip1 in *Saccharomyces cerevisiae*; Asp1 in *Schizosaccharomyces pombe*). The molecular mechanisms of PP-InsP action involve their associating with specific “receptors” [13, 14], the antagonism of PtdIns(3,4,5)P_3_-signaling [15–17], and direct phosphorylation of proteins [18, 19].

Despite the importance of the multifunctional PP-InsPs in signal transduction, it is striking that little is known of the molecular mechanisms that might control PPIP5K and IP6K activities. One recurring mechanism for regulating the signaling functions of kinases comes from cellular control of their access to certain areas of the cell. Moreover, in the specific case of PP-InsPs - no other molecules found in Nature contain such highly-concentrated, three-dimensional phosphate arrays - it has been proposed that determining mechanisms of compartmentation of the synthesis of a particular PP-InsP is key to understanding how each of them may exert a specific function [20]. For example, PP-InsPs are so highly-charged that their non-specific (“delocalized”) electrostatic interactions with proteins may have hindered the evolution of receptor-specificity for a particular PP-InsP, unless it can be compartmentalized [4, 21]. Localized synthesis of an individual PP-InsP could also help prevent the less phosphorylated but more abundant InsP_6_ from competing with binding of that PP-InsP to its receptors [20].

The hPPIP5K1 offers an example of stimulus-dependent compartmentation of PP-InsP turnover. This kinase translocates from the cytoplasm to the plasma membrane in response to activation of PtdIns(3,4,5)P_3_ synthesis [17, 22]. The ensuing, tightly localized catalytic activity of hPPIP5K1 may be a vital mechanism of coincidence detection during activation of PtdIns(3,4,5)P_3_-signaling cascades [17]. In the current study we provide new data that show how hPPIP5K2 exhibits a different mode of compartmentalization.

**Fig. 1:**
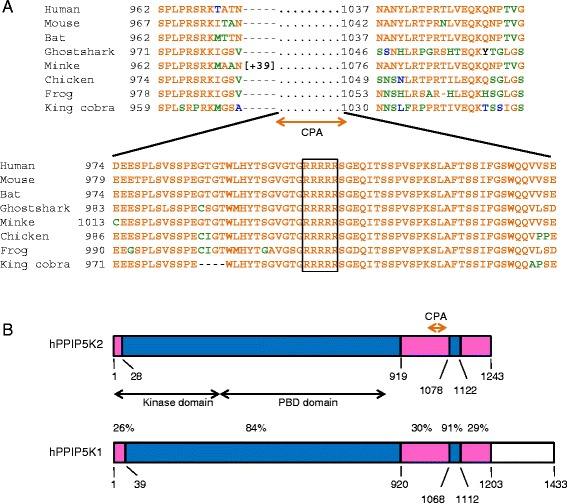
PPIP5K2 but not PPIP5K1 contains a candidate nuclear localization sequence in a highly-conserved context. In Panel **a**, ClustalW was used to generate an alignment of human PPIP5K2 [Swiss-Prot: O43314] with homologues from the mouse, *Mus musculus* [GenBank: XP_006529559]; big brown bat, *Eptesicus fuscus* [Genbank: XP_008142724); minke whale, *Balaenoptera acutorostrata scammoni* [Genbank: XP_007191888.1); ghostshark, *Callorhinchus milii* [GenBank: XP_007891317]; chicken, *Gallus gallus* [GenBank: XP_424859]; frog, *Xenopus tropicalis* [Xenbase: Xetro.A00468.1); king cobra, *Ophiophagus hannah* [GenBank: ETE65579.1]. The candidate nuclear localization sequence (NLS) is boxed. Related amino-acids are grouped into classes - A/G, D/E, F/Y, I/L/V/M, N/Q and S/T - and then color coded based upon their relative prevalence at each position (orange, most prevalent; green, second most prevalent; blue, third most prevalent). Where two groups of amino acids are equally prevalent, priority defaults to alphabetical order. Using these same amino-acid classes, panel **b** depicts the percentage similarities of aligned regions of hPPIP5K2 and hPPIP5K1 [Swiss-Prot: Q6PFW1]; the proteins can be divided into regions that are either >85% similar (coloured blue) or no more than 30% similar (colored pink). The graphics also depict the kinase domains and the PBD (PtdIns(3,4,5)P_3_-binding domains [22]) that are present in both hPPIP5K1 and hPPIP5K2. The position of the 63-residue domain in hPPIP5K2 that Contains Penta-Arginine (CPA) is also indicated; note how it is more conserved than the regions immediately flanking it. PPIP5K1 does not contain sequence that is homologous to the CPA domain, and so is also missing both the penta-Arg NLS and the adjacent Ser residue that is phosphorylated (see text for details)

The amino acid sequence of hPPIP5K2 contains a candidate monopartite nuclear localization signal (NLS): a tri- or tetra-peptide comprising Lys and/or Arg residues [23–25]. This sequence resides within an RRRRR pentapeptide positioned near the C-terminus of hPPIP5K2 (Fig. 1a). Homologues of this isoform that also possess this candidate NLS are distributed throughout much of the animal kingdom (Fig. 1a,b). Furthermore, in each case the penta-Arg lies within a 63-residue sequence that is also highly conserved (Fig. 1a). Neither this sequence, nor the penta-Arg, are conserved in either PPIP5K1 (Fig. 1b), nor in any yeast or invertebrate PPIP5Ks annotated to date. No previous study has addressed whether this PPIP5K2-specific NLS might have any functional significance. The investigation of this possibility has been the main objective of the current study.

We initially used confocal microscopy to study the subcellular distribution of GFP-tagged PPIP5K constructs in HEK293T cells. These experiments demonstrated the existence of a discrete nuclear hPPIP5K2 pool, although its size was relatively small and somewhat variable (see below). It was therefore necessary to study this subcellular compartmentation in a statistically-powerful large number of cells, in an automated manner that eliminates bias in cell selection by the investigator. Two techniques have been used previously for these types of experiments: microscope-based high-content automated image analysis [26] and imaging flow cytometry [27]. These methods also have the benefit of being insensitive to variability in both cell morphology and fluorescence intensity [26–29]. Imaging flow cytometry has the faster throughput, and that is the technique we have employed here. We also used site-directed mutagenesis to establish that the penta-Arg sequence in hPPIP5K2 contributes to the protein’s degree of nuclear localization. Furthermore, mass-spectrometry data and additional mutagenic experiments provided evidence that the functionality of the penta-Arg is influenced by phosphorylation of an adjacent Ser residue. As such, our study provides the first indication that covalent modification of any PPIP5K can alter any aspect of its biological function. Overall, our work offers several new directions for PP-InsP research.

## Methods

### Cell lines and constructs

HEK293T cells were cultured in DMEM supplemented with 10 % FBS at 37 °C in a 5% CO_2_ atmosphere. Gateway cloning was used to prepare expression vectors for tagged versions of hPPIP5K2 [11]: pDEST47/hPPIP5K2 (C-terminally GFP-tagged), and pDEST515/hPPIP5K2 (N-terminally FLAG-tagged). Mutations were introduced using PCR site-directed mutagenesis (Additional file 1: Table S1).

### Mass spectrometry

Neomycin-selected (750 μg/ml; 2 weeks) stable HEK293T cell lines expressing either FLAG-hPPIP5K2 or FLAG-β-galactosidase were plated at a density of 0.5 × 10^6^ cells/60 mm dish in DMEM containing 10 % FBS. After two days, cells were lysed in ice-cold 0.25 ml buffer containing 50 mM HEPES, pH 7.8, 300 mM NaCl, 1.0% NP-40, 10 mM MgSO_4_, 10% glycerol, 10 mM NaF, 1 mM Na_3_VO_4_, protease inhibitor tablets [Roche:11873580001; 1 tablet/10 ml lysis buffer]). Lysates were cleared by centrifugation and then incubated at 0–4 °C with 0.05 volume of ANTI-FLAG® M2 Affinity gel (Sigma: A2220) pre-equilibrated with TBS (50 mM Tris–HCl pH 7.4, and 150 mM NaCl). After 90 min, the resin was centrifuged, washed three times with ice-cold TBS, and bound proteins were eluted with 0.1 volume of 0.1 M glycine-HCl (pH 3.5). Proteins were resolved by SDS-PAGE (NuPage 1.5 mm gels; 45 μl/lane). For study of hPPIP5K2 binding partners, the entire gel lanes were divided into 24 equal sections, each of which were then digested, lyophilized, and resuspended in 40 μl of 0.1% formic acid, prior to analysis. For phosphopeptide analysis of PPIP5K2, the kinase band was excised manually and digested with trypsin (Promega) for 8 h in a Progest robotic digester (Genomic Solutions). Resulting peptides were lyophilized, resuspended in 35 μl of 0.1% formic acid, and enriched using TiO_2_ tips (Glygen) following the manufacturer’s protocol.

Samples were analyzed by nanoLC-ESI-MS and MS/MS, using an Agilent 1100 nanoLC system on-line with an Agilent 6340 ion trap mass spectrometer with the Chip Cube Interface, essentially as described [11]. The Data Extractor feature of the Spectrum Mill software (Agilent) was used to generate peak lists from mass spectroscopy based analysis of peptide digests. A peak list was generated from the data obtained from the nanoLC-ESI-MS/MS analysis using the Data Extractor feature of the Spectrum Mill software from Agilent or the Mascot Distiller software from Matrix Science. The Data Extractor settings included limiting the data search to deconvolved ions observed between 400 and 5000 Da and a retention time between 10 min and 50 min. MS scans with the same precursor mass (+/− 1.5 m/z) and retention time within 30 s were merged. Moreover, of the remaining MS/MS spectra, only those that contained sequence tag information greater than 2 residues were submitted for database searching. The resulting extracted data were then searched against the NCBI or SwissProt/UniProt human databases using the MS/MS Search function in the Spectrum Mill software or the Mascot search engine from Matrix Science. Search settings included a trypsin specificity with one missed cleavage allowed, a precursor ion mass tolerance of 2 Da, a product ion mass tolerance of 0.7 Da, variable methionine oxidation, and a minimum matched spectral intensity of 70%.

### Confocal microscopy

Cells were seeded in eight-well chambered coverglass (Nunc Lab-TekII, Thermo Scientific) for 15 h, and then transiently transfected with 0.5 ug of DNA plasmids using Lipofectamine 3000 (Invitrogen). After 48 h, the DMEM was replaced with Leibovitz’s L-15 medium (Invitrogen). Images were acquired using a Zeiss LSM 780 inverted microscope equipped with a Plan-Neofluar 63 Octovar oil objective. A keypton/argon laser was used for excitation of GFP (488 nm). All images were analysed using Image J software (NIH, Bethesda, MD, USA). To quantify fluorescence intensity of PPIP5K-GFP in the nucleus relative to the cytosol, we measured and averaged fluorescence intensity in 1 pixel areas from three different positions in each compartment. After subtracting background fluorescence intensity, we determined the ratio of fluorescence in the nucleus (F_N_) compared to that in the cytosol (F_C_). The nucleus was identified by Hoechst 33258 staining (Molecular Probes).

### Imaging flow cytometry

Transient transfections were performed in 6- or 12-well plates (0.25 - 0.5 × 10^6^ cells/plate) using either Polyjet (Signagen, Rockville, MD), or FuGENE 6 (Promega, Madison WI). Cells were analyzed 2 days after transfection. For live-cell imaging, cells were harvested into DMEM and washed twice by centrifugation (500 × g; 6 min, room temperature), the second time through a Falcon™ Tube with 35 μm mesh cell strainer cap (Fisher Scientific; Pittsburgh PA); cells were maintained at 37 °C prior to analysis. In other experiments, < 90 min after harvesting, cells were fixed in phosphate-buffered saline (PBS) containing 1% *para*-formaldehyde (37 °C; 10 min), rinsed, and suspended in 120–250 μl PBS prior to analysis. Cell nuclei were stained with 50 μM DRAQ5 (Cell Signaling Technology, Beverly, MA) immediately prior to analysis.

Cell data was acquired at 40X magnification (4 μm depth of field) on a single camera, 6-channel ImageStream^*X*^ flow cytometer (Amnis Corporation, Seattle, WA) using INSPIRE data acquisition software with 488 nm and 658 nm excitation lasers. Brightfield was collected in channel 1. Side-scatter was collected in channel 6 with a 785 nm laser. GFP and DRAQ5 were detected in channel 2 (505–560 nm) and channel 5 (642–745 nm) respectively. Cell classifier was set for a brightfield area lower limit of 50 μm to eliminate debris. Post-acquisition data were analyzed with the nuclear translocation wizard in the IDEAS 5.0 software (Amnis Corporation, Seattle, WA). Individual cells were initially gated by best focus in the DRAQ5 channel, using a gradient root mean square histogram. Single cells were then gated from a plot of brightfield area versus aspect ratio (minor axis/major axis). Double positive cells were gated from the intensity of channel 2 versus intensity of channel 5 dot plot. The degree of cross-correlation of the GFP and DRAQ5 signals was determined using the Similarity Score (log-transformed Pearson’s correlation coefficient) within the IDEAS analysis software on a per-cell basis [28]. Each experiment was performed at least three times.

## Results and Discussion

### Evidence for the functionality of the NLS in hPPIP5K2

**Fig 2:**
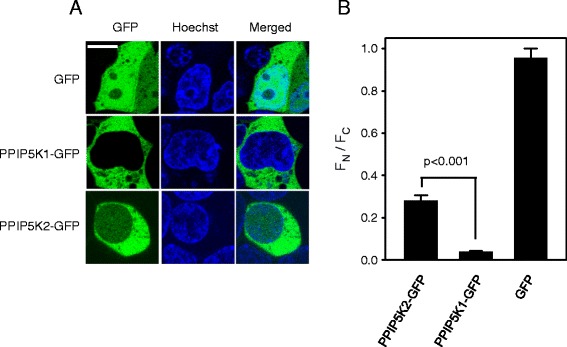
Identification of a nuclear pool of hPPIP5K2. Panel **a** shows confocal microscopy of live HEK293T cells transfected with either GFP alone, hPPIP5K1-GFP, or hPPIP5K2-GFP. Single cells are shown that were selected by-eye to represent approximate median values for the distribution of GFP-signal between nucleus (F_N_) and cytoplasm (F_C_). From each group, fifteen cells were randomly selected and F_N_/F_C_ ratios were calculated (panel **b**). Data shown are means ± SEM (statistical significance was determined using Student's t-test). The size bar represents 10 μm

There are two isoforms of mammalian PPIP5K, types 1 and 2 (Fig. 1 and see [9, 11, 30]); a candidate penta-Arg NLS is specific to hPPIP5K2 (Fig. 1). As a first step towards testing the functionality of the putative NLS, we used confocal fluorescence microscopy to compare the intracellular distribution of hPPIP5K1-GFP and hPPIP5K2-GFP in live HEK293T cells (Fig. 2a,b). We used GFP-tagged constructs because we did not have access to specific antibodies that can be used for imaging endogenous protein. We determined the ratio of fluorescence intensity in the nucleus (F_N_) compared to that in the cytosol (F_C_). The value of the F_N_/F_C_ ratio for hPPIP5K1 is 0.04 (Fig. 2a,b), which indicates that this protein is near-completely excluded from the nucleus. Similar data were obtained in an earlier study [11]. In contrast, a substantially higher F_N_/F_C_ ratio of 0.28 was obtained for hPPIP5K2 (Fig. 2a,b). These data identify an isoform-specific nuclear pool of hPPIP5K2, although it is worth noting the extent of the cell-to-cell variability: the data in Fig. 2b were acquired from 15 cells in which the F_N_/F_C_ ratios varied from 0.16 to 0.5.

**Fig. 3:**
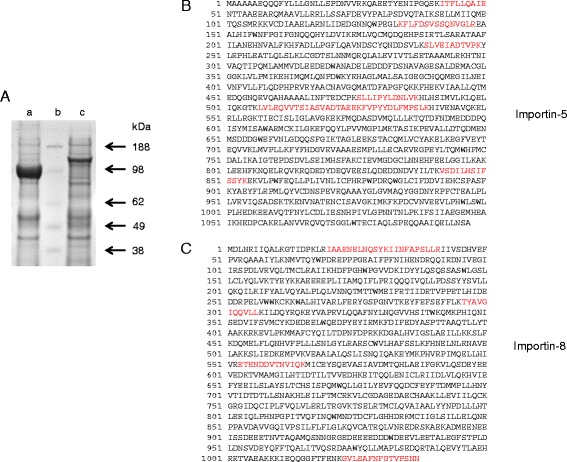
Use of affinity pull-downs and mass spectroscopy to identify Importin-5 and Importin-8 as proteins interacting with hPPIP5K2. Panel **a** shows SDS-PAGE analysis of pull-downs of FLAG-beta-galactosidase (lane a) and FLAG-hPPIP5K2 (lane c), with molecular weight markers (lane b); see the Methods Section for further details. The entire gel lanes (a and c) were divided into 24 equal sections, each of which were then digested, lyophilized, and resuspended in 40 μl of 0.1% formic acid, prior to analysis. Panel **b** shows that importin-5 was identified in 13 spectra from 7 unique peptides (red font) and a distinct summed MS/MS search score of 91.06, yielding 8.2% sequence coverage. Panel **c** shows that importin-8 was identified by 6 spectra from 5 unique peptides (red font) and a distinct summed MS/MS search score of 68.63 with 5.9% sequence coverage. The individual spectra are provided in Additional file 2: Figures S1 and S2

NLSs interact with α/β-importin complexes that shuttle many nuclear proteins into the nucleus immediately after their translation [31]. Other proteins - particularly those that participate in cell signaling - may not have a predominantly nuclear localization by default, but instead have their nuclear delivery regulated by certain β-importins that act independently of α-importin [32]. Thus, the identification of importins that associate with novel cargoes can be functionally illuminating [33]. We searched for importins that might associate with hPPIP5K2 by using a subtractive proteomic approach: either FLAG-tagged hPPIP5K2, or an epitope control, FLAG-tagged β-galactosidase, were stably expressed in HEK293T cells. Lysates were prepared, and incubated with immobilized anti-FLAG antibodies to pull-down the respective baits and associated proteins (Fig. 3a), which were then identified by mass-spectrometry (Fig. 3b,c; Additional file 2: Figures S1 and S2). Importin-5 was identified in 13 spectra from 7 unique peptides yielding 8.2% sequence coverage (Fig. 3b). Importin-8 was identified by 6 spectra from 5 unique peptides with 5.9% sequence coverage (Fig. 3c). Neither of these importins were identified in the control pull-downs with FLAG-galactosidase. Importin-5 and importin-8 are members of the β-importin family that are receiving increasing attention for regulating nuclear delivery of certain signaling proteins [32]. These data are consistent with the conclusion (see above) that hPPIP5K2 has some capacity to undergo nuclear translocation.

### Mutagenesis of the NLS in hPPIP5K2 inhibits its nuclear translocation

We next investigated if the degree of nuclear localization of hPPIP5K2 would be inhibited by site-directed mutagenesis of the enzyme’s putative NLS. Faced with the challenge that the nuclear pool of hPPIP5K2 is relatively small and variable (see above), we considered it imperative to collect data from a statistically-powerful large number of cells, in an automated manner that eliminates bias in cell selection by the investigator. Imaging flow cytometry is a technique that can meet these requirements [27–29].

As an alternative to using F_N_/F_C_ ratios to quantify relative nuclear localization of a protein (as in the data described by Fig. 2), imaging flow cytometry performs an alternative but widely-used analytical method: co-localization analysis. Here, a specific region of the cell is digitally isolated - “masked” - and then a pixel-by-pixel determination is made of the degree of correlation between two spectrally distinct signals in that particular region of interest. So, for example, the degree of co-localization of a GFP-tagged protein and a DRAQ5 nuclear stain, tallies with the extent of nuclear accumulation of that particular protein. Pearson’s Correlation Coefficient has been used frequently to quantify the degree of co-localization between two spectral signals [34]. More recently [28], a log transformation of Pearson’s Correlation Coefficient - the Similarity Score - was introduced. The latter has a wider dynamic range that correlates more closely to qualitative judgments of visual distinctiveness [28].

**Fig. 4:**
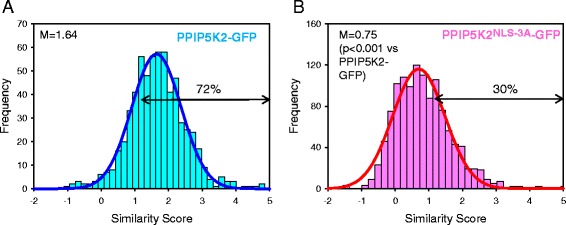
Analysis by imaging flow cytometry of the degree of nuclear localization of hPPIP5K2-GFP and hPPIP5K2^NLS-3A^-GFP. Live HEK293T cells, transfected with either **a**, hPPIP5K2-GFP (n = 632; 44236 ± 1711 total cellular fluorescence units) or **b**, hPPIP5K2^NLS-3A^-GFP (n = 1195, 48900 ± 1367 total cellular fluorescence units), and treated with the nuclear DRAQ5 stain, were analyzed for the degree of co-localization of the two spectral signals in every nuclear pixel (approx. 700 per cell), using imaging flow cytometry. The histograms depict the frequency of binned Similarity-Scores [28]; best-fit Gaussian plots are added. The horizontal arrows highlight the percentage of cells in which the expressed hPPIP5K2 construct was determined to have a significant nuclear localization (Similarity Score > 1.2; [35, 36]). The median value (M) of the Similarity Score for cells expressing hPPIP5K2^NLS-3A^-GFP is significantly lower than that for cells expressing WT enzyme (p < 0.001; Mann–Whitney Rank Sum Test)

We used imaging flow cytometry to quantify the degree of co-localization of hPPIP5K2-GFP and DRAQ5 in individual HEK293T cells. The Similarity Scores were calculated from an analysis of each of the approximately 700 individual pixels covering every nucleus (Fig. 4a). In a representative experiment with 632 live cells, the population of Similarity Scores were distributed normally, as expected for a biological phenomenon. Thus, the scores from approximately 2/3 of all cells were within one standard deviation of the mean (Fig. 4a). The median value of the Similarity Score of this is cell population was 1.64 (Fig. 4a). A score >1.2 designates co-localization of two spectral signals [35, 36]; this criterion was met by 72% of all of the cells that contributed to the data described in Fig. 4a. Thus, the data obtained by imaging flow cytometry (Fig. 4a) provide statistically robust confirmation of the conclusion drawn from confocal imaging (Fig. 2) that cells contain a distinct nuclear pool of hPPIP5K2.

We next tested the functionality of the putative NLS by creating a construct (i.e. hPPIP5K2^NLS-3A^-GFP), in which the penta-Arg sequence (Fig. 1a) was mutated to RAAAR. In HEK293T cells expressing hPPIP5K^NLS-3A^-GFP, the median value for the similarity score was 0.75 (Fig. 4b). Since the Similarity Score is a logarithmic function, our data indicate that the NLS mutant exhibits 3.6-fold less nuclear co-localization with DRAQ5 than did the WT hPPIP5K2-GFP (p < 0.001). These data provide the first direct demonstration of the functionality of this NLS. Note that 30% of cells expressing hPPIP5K^NLS-3A^-GFP exhibited some residual nuclear co-localization with DRAQ5 (Fig. 4b). Thus, even though our data indicate that the canonical NLS makes a major contribution to the extent of nuclear localization of hPPIP5K2, we do not exclude that other factors may also be involved.

**Fig. 5:**
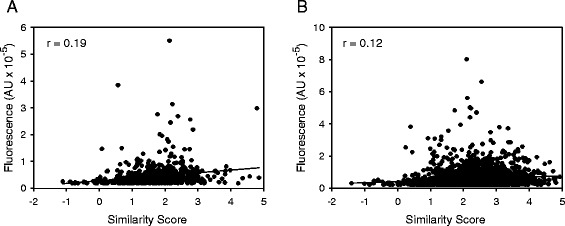
The degree of nuclear localization of hPPIP5K2-GFP is independent of its degree of expression. Either (panel **a**) live, or (panel **b**) fixed, HEK293T cells that had been transfected with hPPIP5K2-GFP were treated with the nuclear DRAQ5 stain. The scatter plots show the total GFP fluorescence in each individual cell plotted against the same cell’s Similarity Score. The values of the correlation coefficients (r) are shown in each panel

The mean values of total cellular fluorescence of the hPPIP5K2-GFP and hPPIP5K2^NLS-3A^-GFP cell populations were very similar (legend to Fig. 4), indicating comparable levels of construct expression (and see below). Nevertheless we also investigated, on a cell-by-cell basis, whether the total cellular fluorescence exhibited any correlation with the Similarity Score for nuclear localization (Fig. 5). The values of the correlation coefficients for this comparison are close to zero (Fig. 5). That is, we make the important observation that the degree of nuclear localization of the kinase was not influenced by variations in its overall degree of expression.

### Nuclear localization of hPPIP5K2 is regulated by phosphorylation of S1006

**Fig. 6:**
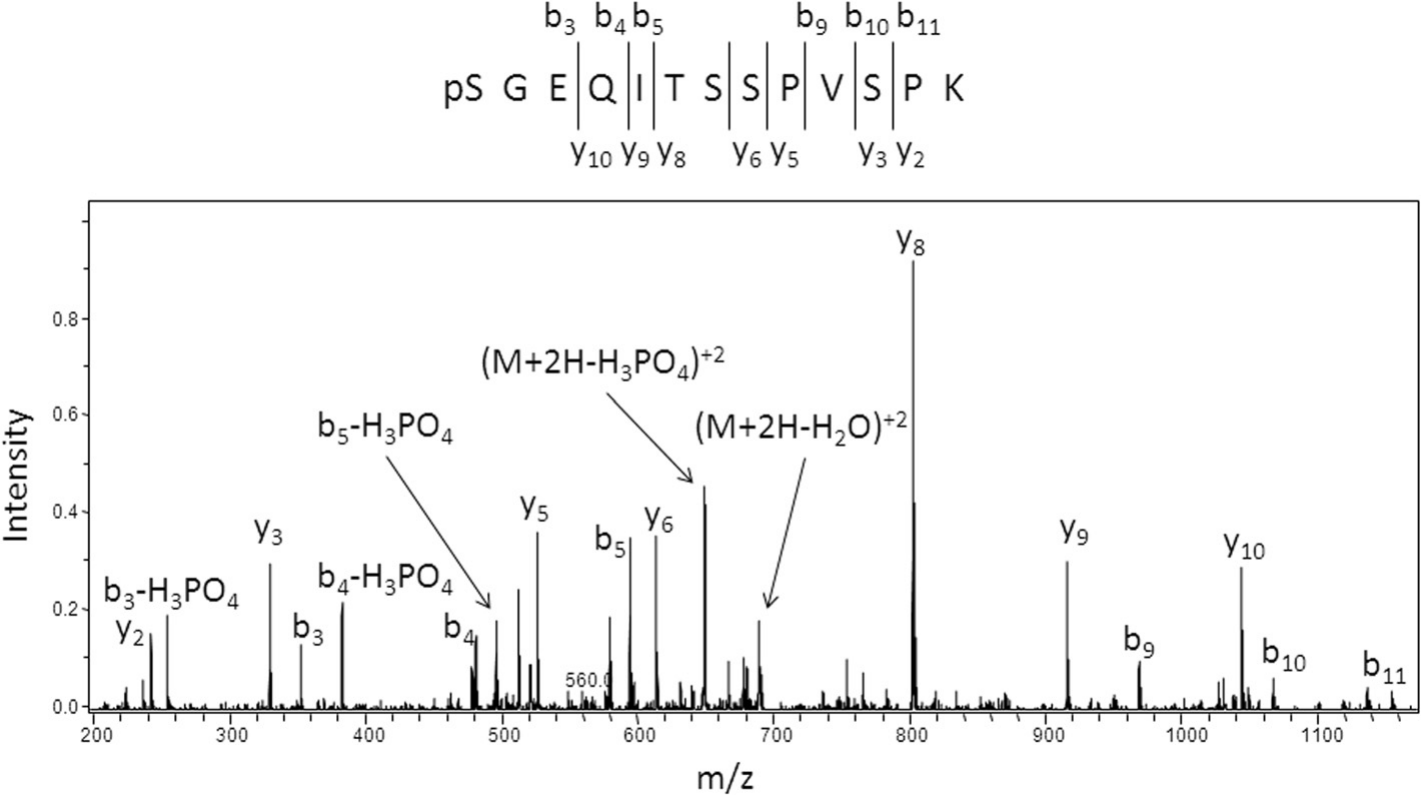
Identification of phospho-S1006 in hPPIP5K2 by mass spectroscopy. Tandem MS spectrum of a doubly charged ion at m/z 698.7 corresponding to the phosphorylated form of the hPPIP5K2 peptide spanning residues 1006–1018 (SGEQITSSPVSPK). Extensive b- and y-ion series unambiguously identify this peptide and also localize the site of phosphorylation to the N-terminus of the peptide at S1006

There are a number of examples of phosphorylation/dephosphorylation of proteins regulating their trafficking between the nucleus and cytoplasm [37]. For example, the addition of a negatively charged phosphate group close to an NLS can interfere with its electrostatic interactions with importins [38, 39]. In the case of the adenomatous polyposis coli protein [40], the function of its monpartite NLS is inhibited upon phosphorylation of a proximal Ser residue (i.e. in the context of PKKKRPS). A conserved Ser residue (S1006) is present immediately C-terminal of the NLS in hPPIP5K2 (i.e., RRRRRS; Fig. 1a). We therefore investigated if this Ser might be phosphorylated and impact the nuclear localization of hPPIP5K2. The FLAG-tagged PPIP5K2 that we expressed in HEK293T cells (see above), was pulled-down using anti-FLAG beads, purified by gel electrophoresis, and then interrogated for phosphorylated residues by nanoLC-ESI-MS and MS/MS. This approach identified phospho-Ser at position 1006 (Fig. 6).

We interrogated the sequence around S1006 using PhosphoMotif [41], to search for consensus motifs that might give clues as to the identity of the protein kinase responsible for phosphorylation of this residue. However, the penta-Arg sequence immediately N-terminal of S1006 satisfies the consensus for at least ten different protein kinases. Additional research is therefore required in order to establish which physiological conditions might lead to modification of the degree of phosphorylation of S1006.

**Fig. 7:**
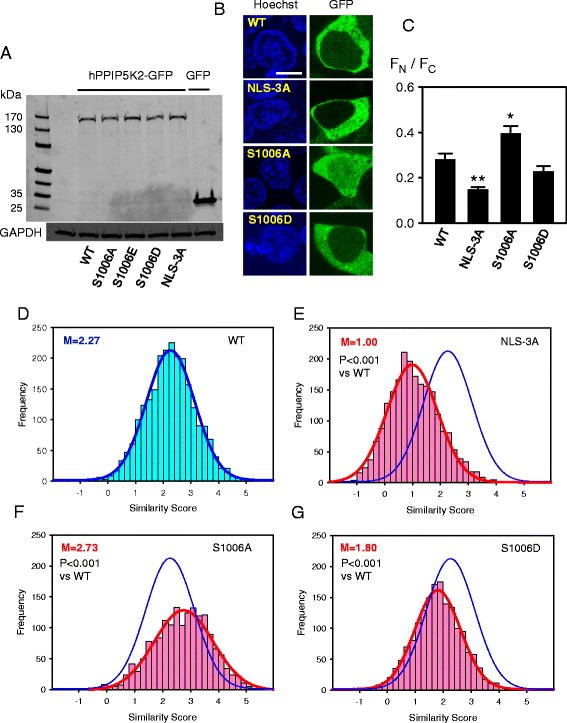
The effects of mutating either the NLS or the adjacent S1006 upon the degree of nuclear localization of hPPIP5K2. Panel **a** shows Western analysis of extracts of HEK293T cells in which various hPPIP5K2-GFP constructs were expressed. For clarity, only four of the molecular markers are labeled. Each of the lanes transfected with a hPPIP5K-GFP construct showed a faint immunoreactive band that corresponded to the migration of GFP alone. ImageJ analysis indicated this represented <2% of the total signal, which is too small to compromise any of our conclusions. Panel **b** shows representative images of live HEK293T cells that expressed each of the various constructs. The size bar represents 10 μm. From each group, fifteen cells were randomly selected and F_N_ / F_C_ ratios were calculated (panel **c**); data represent means ± SEM (** p < 0.001, *p < 0.02 compared to wild-type (WT), determined using Student's t-test). Imaging flow cytometry was also used to analyze fixed and DRAQ5 stained HEK293T cells that were transfected with either **d**, hPPIP5K2-GFP (n = 2357; 57719 ± 1116 total fluorescence units); **e**, hPPIP5K2^NLS-3A^-GFP (n = 2179; 52386 ± 907 total fluorescence units); **f**, hPPIP5K2^S1006A^-GFP (n = 1686; 50383 ± 947 total fluorescence units); **g**, hPPIP5K2^S1006D^-GFP (n = 1696; 57991 ± 1162 total fluorescence units). The histograms depict the frequency of binned. Similarity Scores denoting the degree of co-localization of the GFP signal and nuclear stain, derived on a cell-by-cell basis. Best-fit Gaussian plots are also shown. The blue Gaussian plot for hPPIP5K2-GFP is superimposed upon the other plots for comparative purposes. Statistical differences in median similarity scores (“M”) compared to hPPIP5K2-GFP were determined using a Mann–Whitney Rank Sum Test. Note that the Western analysis includes an extract from cells transfected with a hPPIP5K2^S1006E^-GFP construct. The Similarity Score of the latter (not shown) was not significantly different from that of hPPIP5K2^S1006D^-GFP

We used a mutagenic approach to investigate whether the phosphorylation status of S1006 impacts nuclear localization (Fig. 7a). For illustrative purposes, we also added to these experiments an analysis by traditional confocal microscopy. The small number of cells analyzed (n = 15 for each group; Fig. 7b,c) make these qualitative rather that quantitative observations. Nevertheless, these data show that the hPPIP5K2^NLS-3A^ mutant exhibited significantly less nuclear localization than did wild-type enzyme. We also analyzed the distribution of a hPPIP5K2^S1006A^ mutant in which S1006 was rendered non-phosphorylatable; this mutant showed a significantly higher degree of nuclear localization than did wild-type enzyme (Fig. 7b,c). Such data indicate that the phosphorylation of S1006 that we have observed (Fig. 6) normally restricts the degree of translocation of hPPIP5K into the nucleus. Nevertheless, as described above, a far more rigorous analysis can be accomplished by acquiring data from a large number of cells, in an automated manner that eliminates the possibility of investigator bias. We therefore quantified the nuclear localization of these mutants by Similarity Scores, obtained by imaging flow cytometry. We used fixed cells to facilitate larger sample sets.

**Fig. 8:**
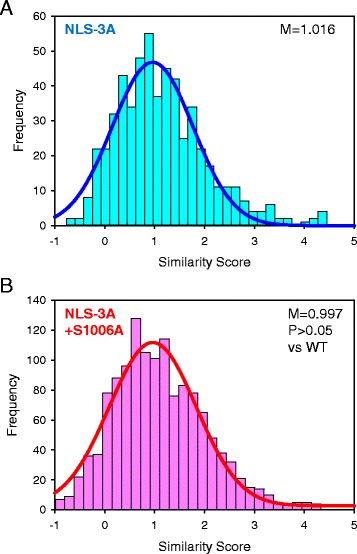
Comparison of the degree of nuclear localization of hPPIP5K2^NLS-3A^-GFP and hPPIP5K2^NLS-3A/S1006A^-GFP. Shown is an analysis by imaging flow cytometry of HEK293T cells that were transfected with either **a**, hPPIP5K2^NLS-3A^-GFP (n = 555; 75893 ± 3073 total fluorescence units) or **b** hPPIP5K2^NLS-3A/S1006A^-GFP (n = 1328; 83844 ± 1716 total fluorescence units), and then stained with DRAQ5. The histograms depict the frequency of binned. Similarity Scores denoting the degree of co-localization of the GFP signal and nuclear stain, derived on a cell-by-cell basis. Best-fit Gaussian plots are also shown

In a representative experiment, the median Similarity Score for the hPPIP5K2^NLS-3A^ mutant (1.00) was confirmed to be significantly lower (p < 0.001) than that for wild-type enzyme (2.27) (Fig. 7d,e). The hPPIP5K2^S1006A^ mutant (in which residue 1006 was non-phosphorylatable) exhibited a highly significant (P < 0.001) increase in the median value of the Similarity Score (Fig. 7d,f). These data indicate a 58% higher degree of nuclear localization of hPPIP5K2^S1006A^ compared to WT enzyme, since the Similarity Score is a logarithmic function. These data provide a quantitatively reliable context to the qualitative observations described in Fig. 7b,c. As such, the data obtained by imaging flow cytometry validate our novel conclusion that phosphorylation of S1006 limits translocation of hPPIP5K into the nucleus. The specificity of this effect of the S1006A mutation is supported by further experiments in which it did not alter the Similarity Score in the hPPIP5K2^NLS-3A^ background, in which the NLS itself is rendered non-functional (Fig. 8). Thus, the entry of hPPIP5K2 into the nucleus that we have observed is a regulated event, rather than a non-specific phenomenon. Overall, our data provide the first indication that covalent modification of any PPIP5K can alter its biological function.

We also constructed a S1006D phosphomimetic mutant of hPPIP5K2. Compared to WT kinase, the hPPIP5K^S1006D^ mutant exhibited a highly significant (P < 0.001) decrease in its Similarity Score, indicative of a 40% reduction in its degree of nuclear localization (Fig. 7d,g). A similar trend was observed in a much smaller number of cells analyzed by traditional confocal microscopy, although in this case the difference from WT enzyme did not attain statistical significance (Fig. 7b,c). These data underscore the biological value of analyzing a large number of cells, in an unbiased and automated manner, when studying the regulation of a sub-pool of a cellular protein.

## Conclusions

The current study describes for the first time a functional NLS in the conserved PPIP5K2 family. We have further demonstrated that, in the case of hPPIP5K2, there is phosphorylation of a Ser residue that is proximal to the NLS. The addition of a negatively charged phosphate group close to an NLS can interfere with its electrostatic interactions with importins [38, 39]. By mutagenesis of this Ser residue, we obtained evidence that its phosphorylation reduces the degree of nuclear localization of this kinase. Our new data advance our insight into compartmentation of PP-InsP synthesis, and also provide the first indication that covalent modification of a PPIP5K may regulate its function.

There has been limited insight into consensus sequences for functional NLSs, and phosphorylation events that regulate NLS function [23, 42]. Thus, there is a continuing need to experimentally resolve the amino-acid residues that define both nuclear trafficking and its regulation. Our accomplishments should contribute to improving the overall predictive reliability of NLS algorithms.

## Additional files

Additional file 1: Table S1. Mutagenic primers for mutagenesis of hPPIP5K2. Additional file 2: Figure S1. Identification of importin-5 as a binding partner of hPPIP5K2. Panels a-g show the individual MS/MS spectra from which the data shown in Fig. 3b were obtained. **Figure S2.** Identification of importin-8 as a binding partner of hPPIP5K2. Panels** a-e** show the individual MS/MS spectra from which the data shown in Fig. 3c were obtained.

## Abbreviations

CPA: Contains penta-arginine
PBS: Phosphate-buffered saline
NLS: Nuclear localization signal
TBS: Tris-buffered saline
