# Supplementary material for: Identification of a functional nuclear translocation sequence in hPPIP5K2
Source: BMC Cell Biol. 2015 Jun 18;16:17. doi: 10.1186/s12860-015-0063-7 (PMC4472268; doi:10.1186/s12860-015-0063-7)
Supplement: Additional file 1: Table S1. — Mutagenic primers for mutagenesis of hPPIP5K2. [file 12860_2015_63_MOESM1_ESM.doc]

**Supplemental Information**

**Table S1. Mutagenic primers for mutagenesis of hPPIP5K2**

S1006A: CGAAGACGCAGAGCGGGGGAACAAATCACTTCTTCC

S1006D: CGAAGACGCAGAGACGGGGAACAAATCACTTCTTCC

S1006E: CGAAGACGCAGAGAGGGGGAACAAATCACTTCTTCC

NLS3A: GCATTATACCAGTGGTGTGGGTACTGGGCGTGCGGCAGCAAGATCAGGGGAACAAATCACTTCTTCCC

The 5'-versions of the complementary primers are shown. Mutagenic nucleotides are underlined.

**Figure S1. Identification of importin-5 as a binding partner of hPPIP5K2.**

Panel A shows SDS-PAGE analysis of pull-downs of FLAG-beta-galactosidease (lane a) and FLAG-hPPIP5K2 (lane c), with molecular weight markers (lane b); see the Methods Section in the main text for further details. The entire gel lanes (a and c) were divided into 24 equal sections, each of which were then digested, lyophilized, and resuspended in 40 μl of 0.1% formic acid, prior to analysis. The Data Extractor feature of the Spectrum Mill software (Agilent) was used to generate peak lists from mass spectroscopy based analysis of peptide digests. A peak list was generated from the data obtained from the nanoLC-ESI-MS/MS analysis using the Data Extractor feature of the Spectrum Mill software from Agilent or the Mascot Distiller software from Matrix Science. The Data Extractor settings included limiting the data search to deconvolved ions observed between 400 and 5000 Da and a retention time between 10 minutes and 50 minutes. MS scans with the same precursor mass (+/- 1.5 m/z) and retention time within 30 seconds were merged. Moreover, of the remaining MS/MS spectra, only spectra that contained sequence tag information greater than 2 residues were submitted for database searching. The resulting extracted data were then searched against the NCBI or SwissProt/UniProt human databases using the MS/MS Search function in the Spectrum

Mill software or the Mascot search engine from Matrix Science. Search settings included a

trypsin specificity with one missed cleavage allowed, a precursor ion mass tolerance of 2 Da, a

product ion mass tolerance of 0.7 Da, variable methionine oxidation, and a minimum matched

spectral intensity of 70%. Panel A shows that importin 8 was identified in 6 MS/MS spectra

covering 5 unique peptides and a distinct summed MS/MS search score of 68.63 with 5.9%

sequence coverage. Panels B-I show the individual MS/MS spectra.

**Figure S2. Identification of importin-8 as a binding partner of hPPIP5K2.**

The Data Extractor feature of the Spectrum Mill software (Agilent) was used to generate peak

lists from mass spectroscopy based analysis of peptide digests of proteins that co-associate with

hPPIP5K2 in pull-down assays. The resulting extracted data were then searched against the

NCBI non-redundant database as described in the legend to supplementary Figure S1. Panel A

shows that importin-8 was identified in 6 spectra covering 5 unique peptides and a distinct

summed MS/MS search score of 68.63 with 5.9% sequence coverage (red font). Panels B-F

show the individual spectra.
