# Supplementary material for: Identification of a functional nuclear translocation sequence in hPPIP5K2
Source: BMC Cell Biol. 2015 Jun 18;16:17. doi: 10.1186/s12860-015-0063-7 (PMC4472268; doi:10.1186/s12860-015-0063-7)

Figure S1A

Importin-5

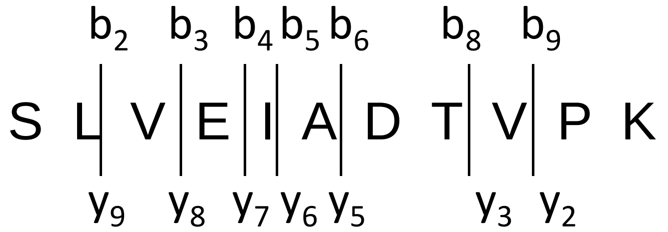

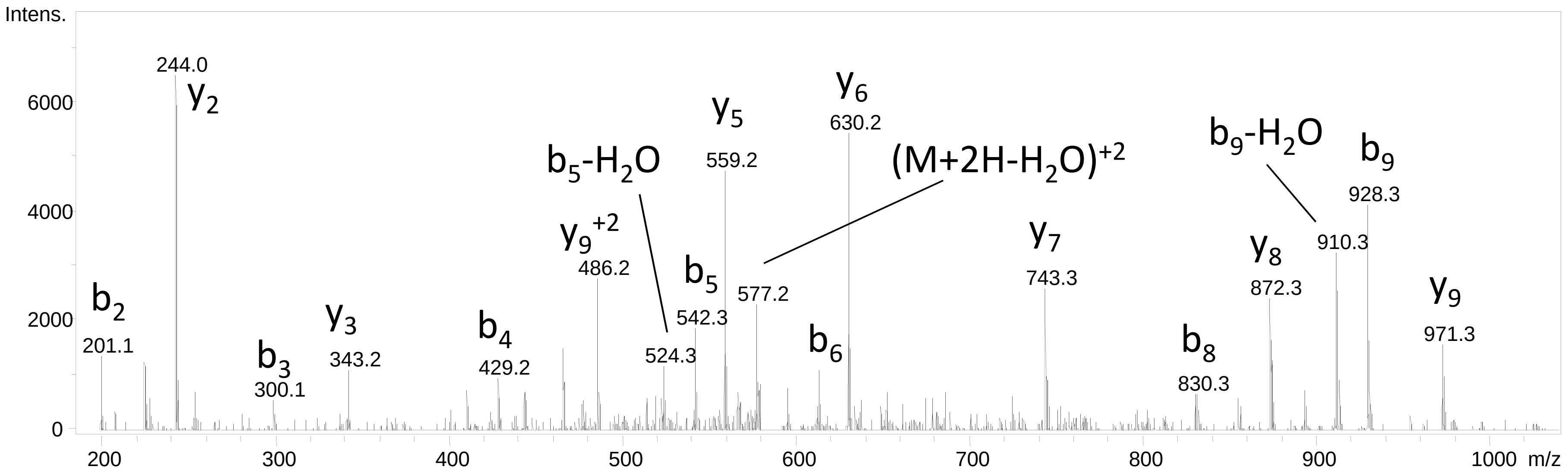

Figure S1B

Importin-5

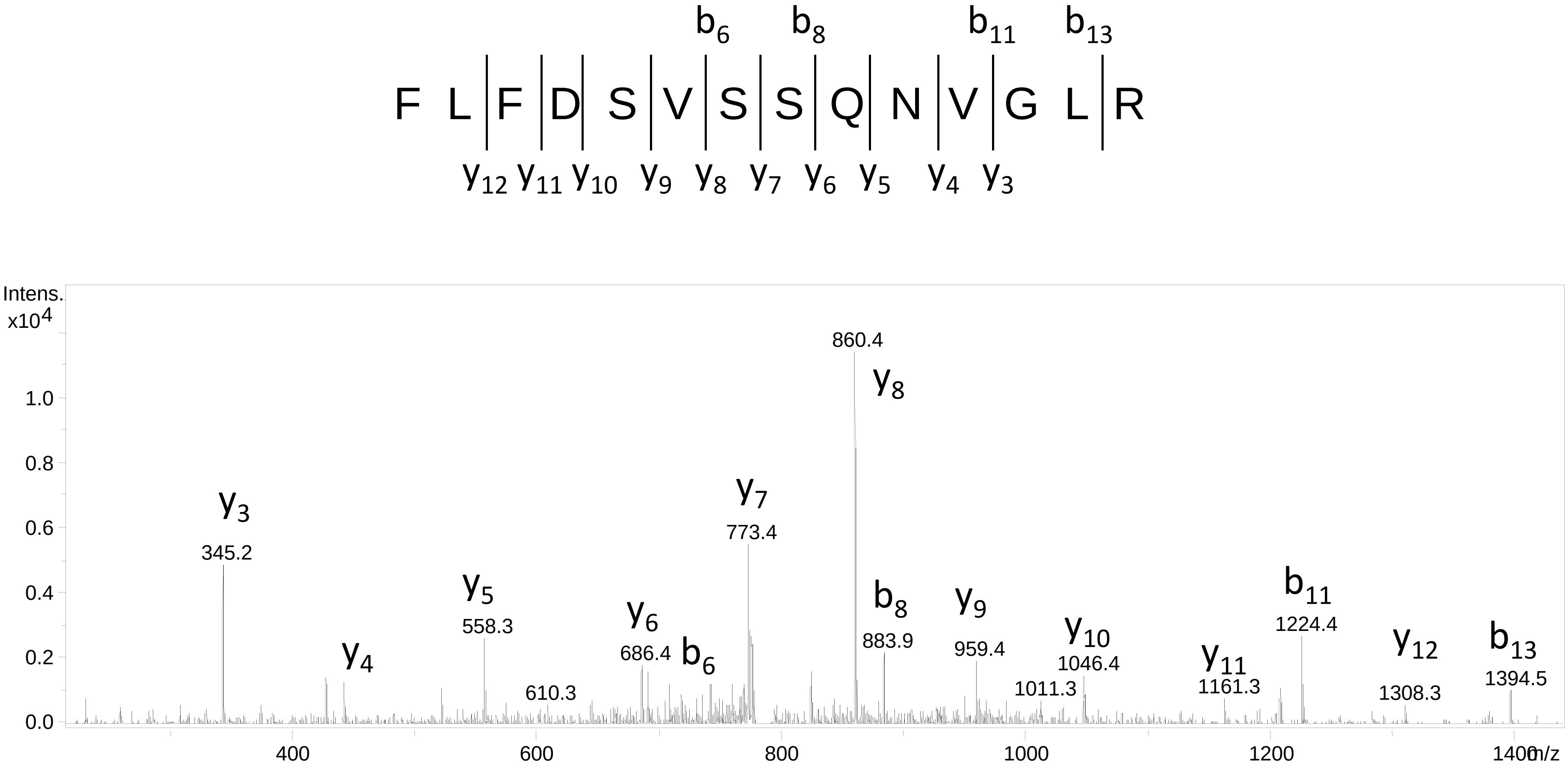

Figure S1C

Importin-5

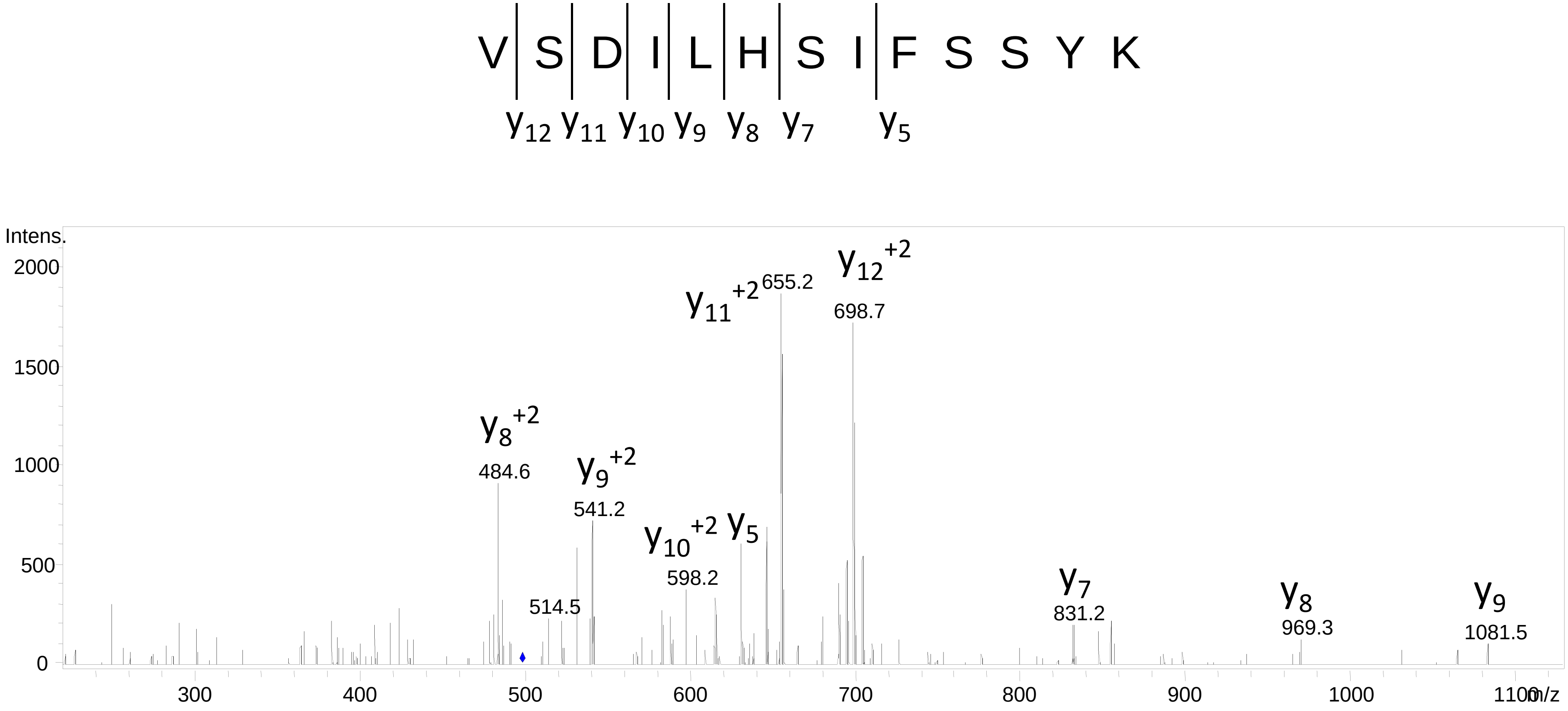

Figure S1D

Importin-5

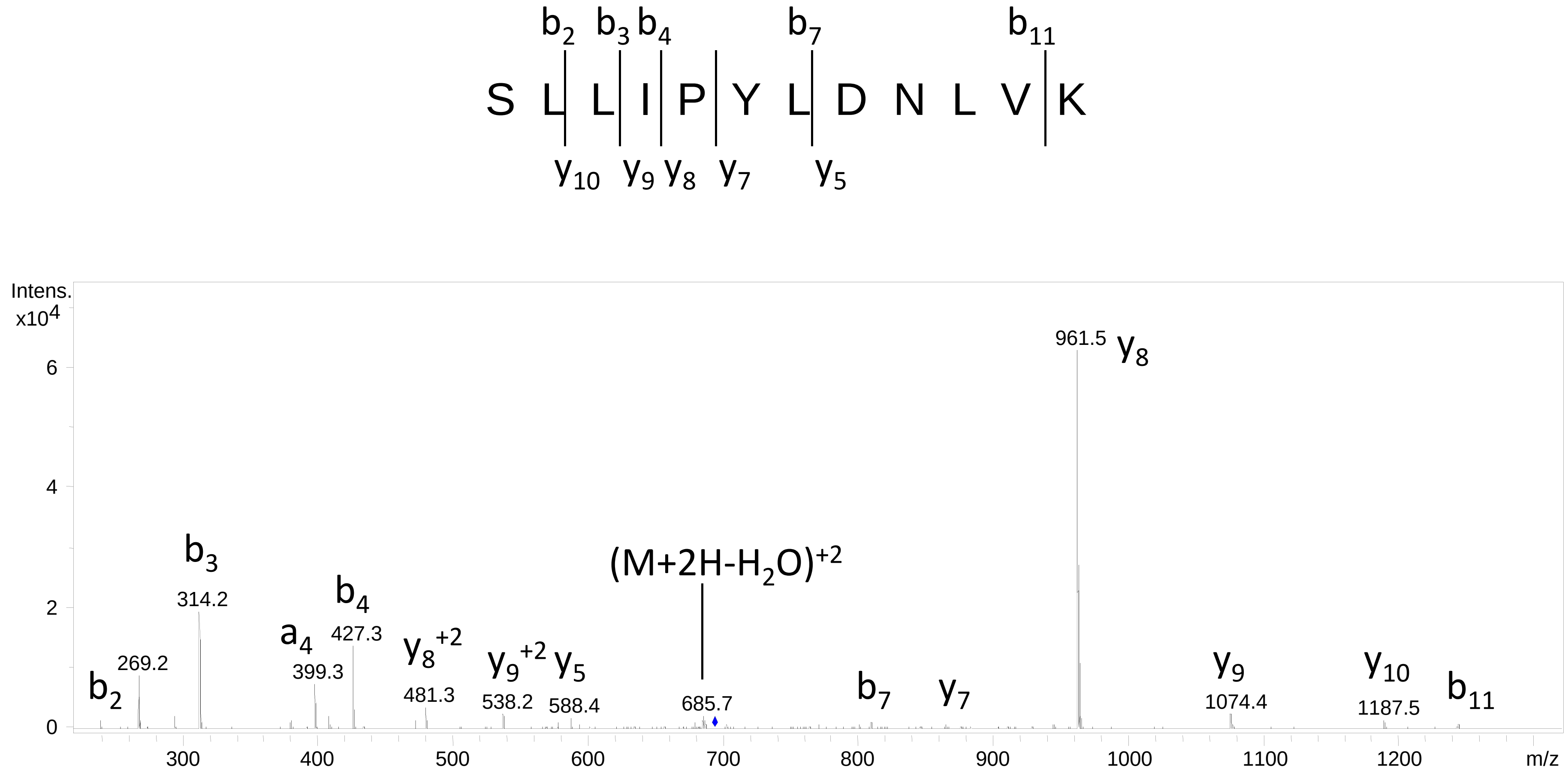

Figure S1E

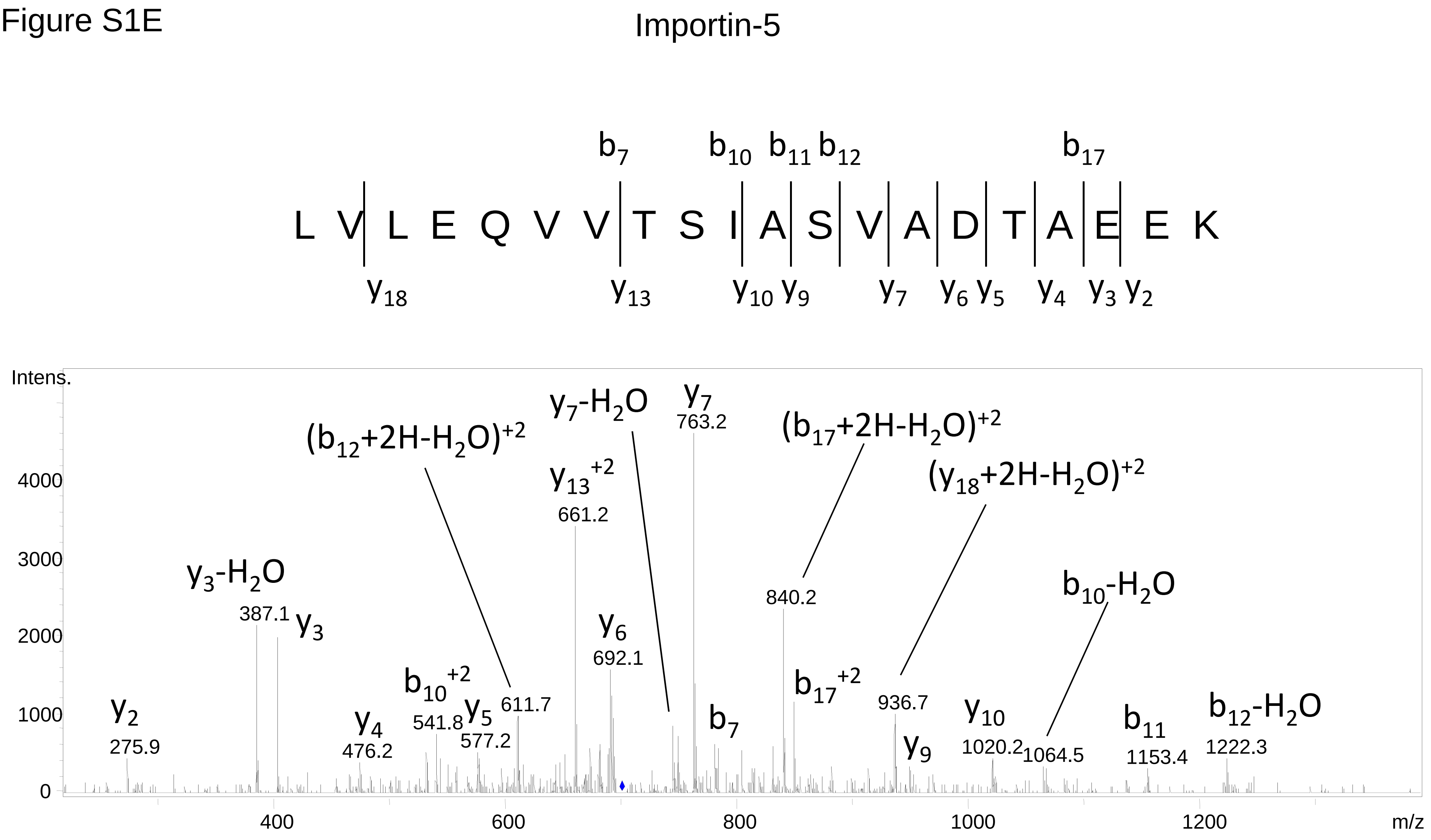

Importin-5
b7
b10
b11
b12
b17
L V L E Q V V T S I A S V A D T A E E K
y18
y13
y10
y9
y7
y6
y5
y4
y3
y2
Intens.
4000
3000
2000
1000
0
400
600
800
1000
1200
m/z
y2
275.9
y3-H2O
387.1
y3
y4
476.2
b10+2
541.8
y5
577.2
(b12+2H-H2O)+2
611.7
y13+2
661.2
y6
692.1
y7-H2O
y7
763.2
b7
(b17+2H-H2O)+2
840.2
b17+2
(y18+2H-H2O)+2
936.7
y9
y10
1020.2
b10-H2O
1064.5
b11
1153.4
b12-H2O
1222.3

Figure S1F

Importin-5

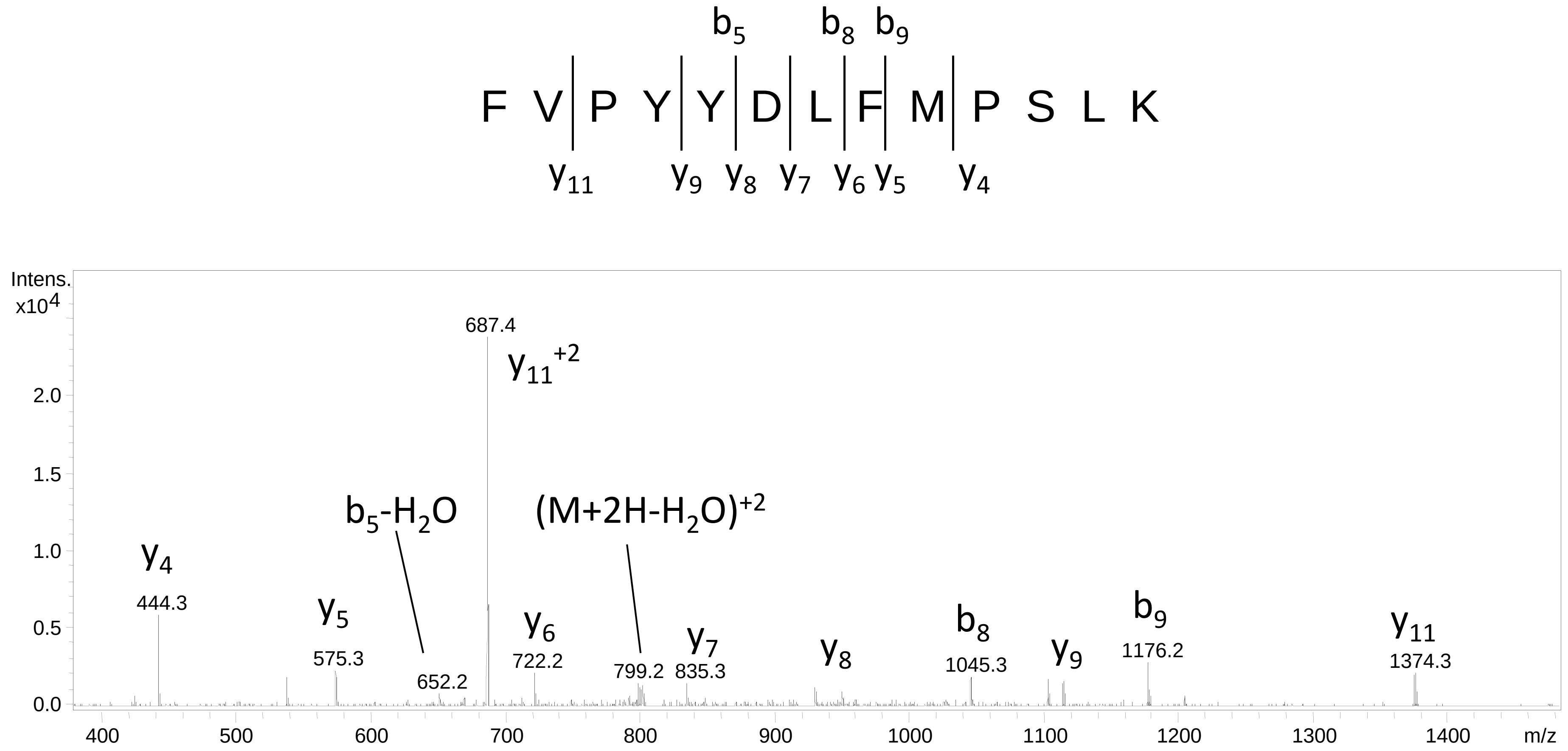

Figure SIG

Importin-5

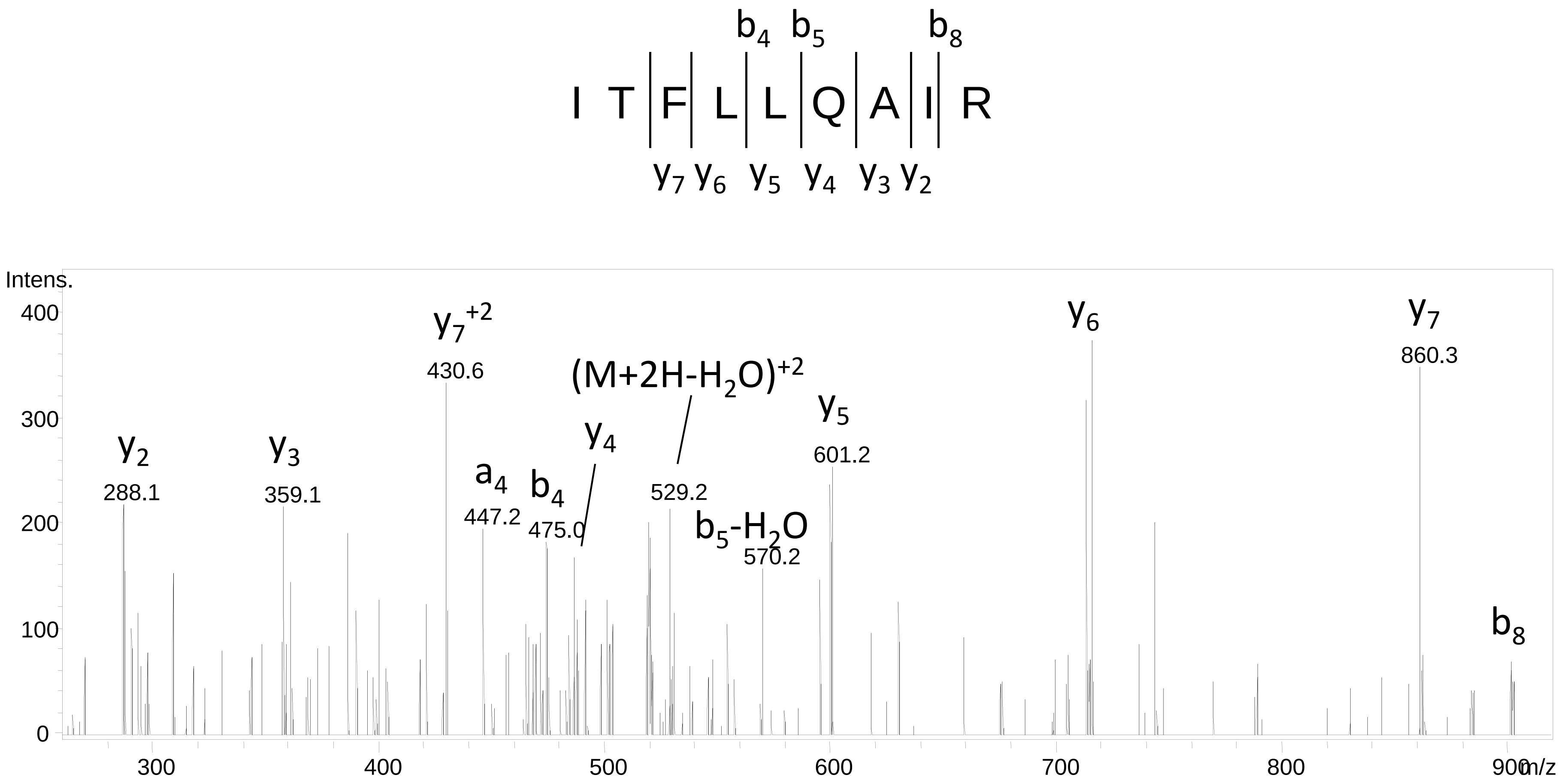

Figure S2A

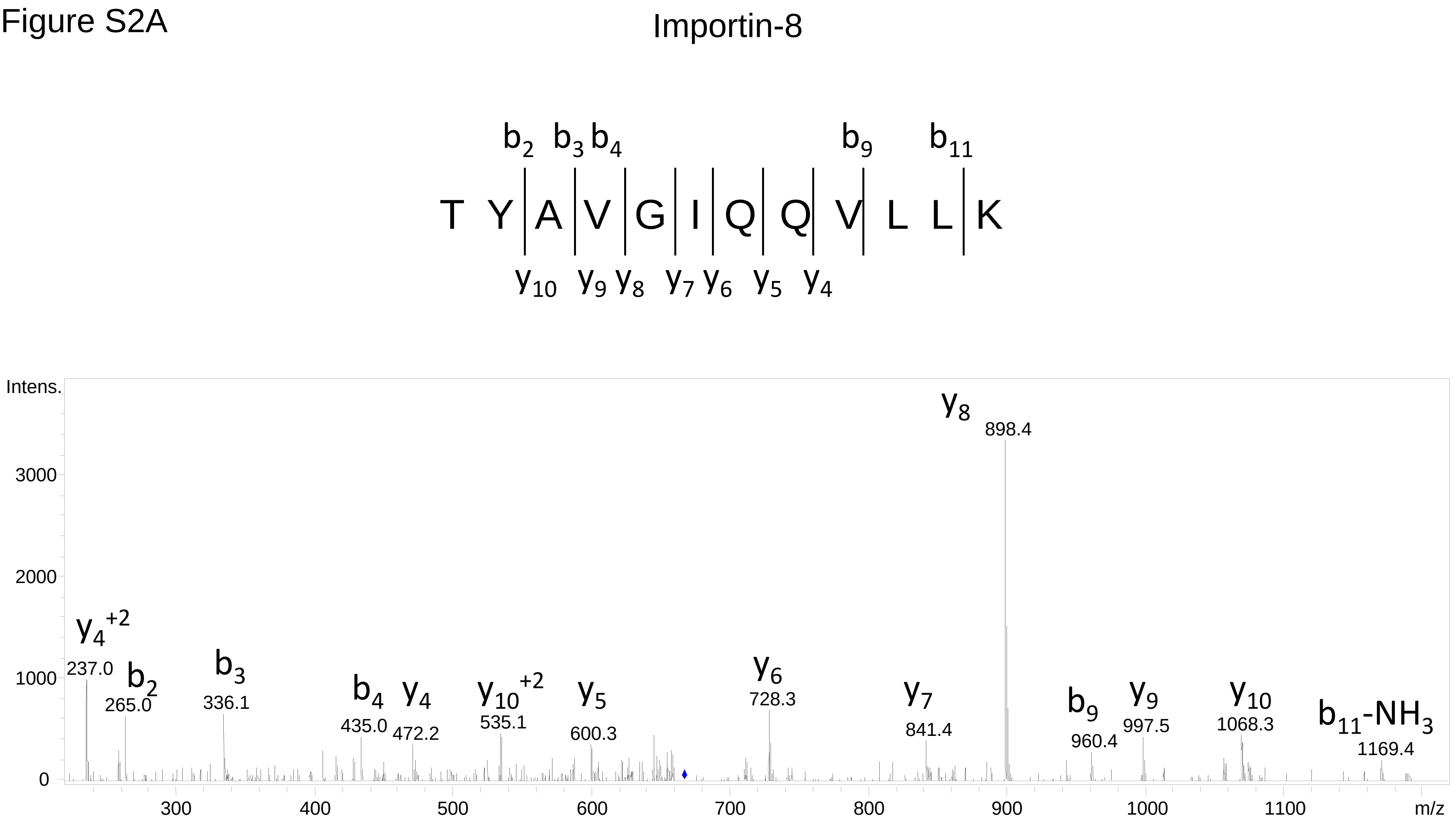
Importin-8
b2 b3 b4 b9 b11
T Y A V G I Q Q V L L K
y10 y9 y8 y7 y6 y5 y4
Intens.
3000
2000
1000
0
y8
898.4
y4+2
237.0
b2
265.0
b3
336.1
b4
435.0
y4
472.2
y10+2
535.1
y5
600.3
y6
728.3
y7
841.4
b9
960.4
y9
997.5
y10
1068.3
b11-NH3
1169.4
300
400
500
600
700
800
900
1000
1100
m/z

Figure S2B

Importin-8

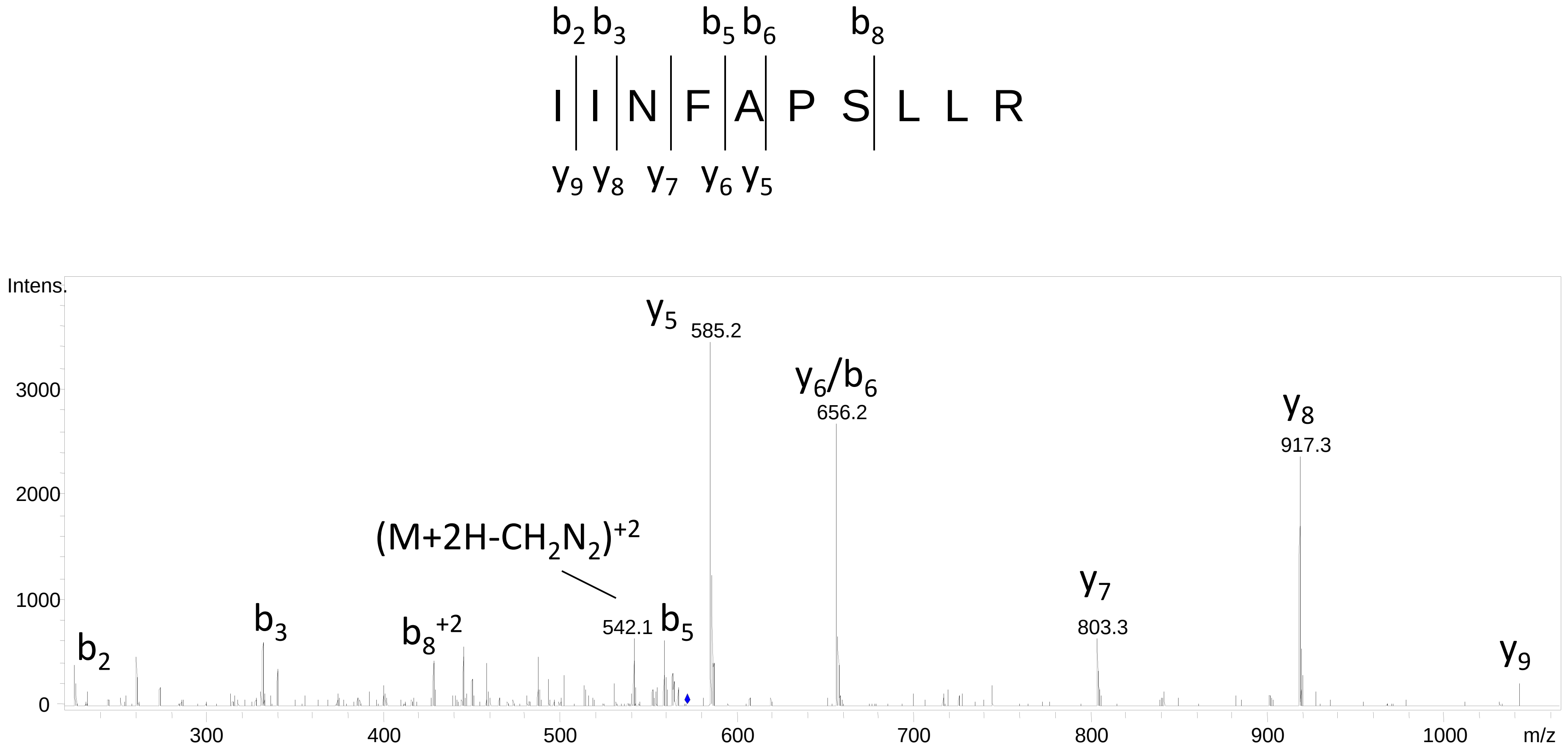

Figure S2C

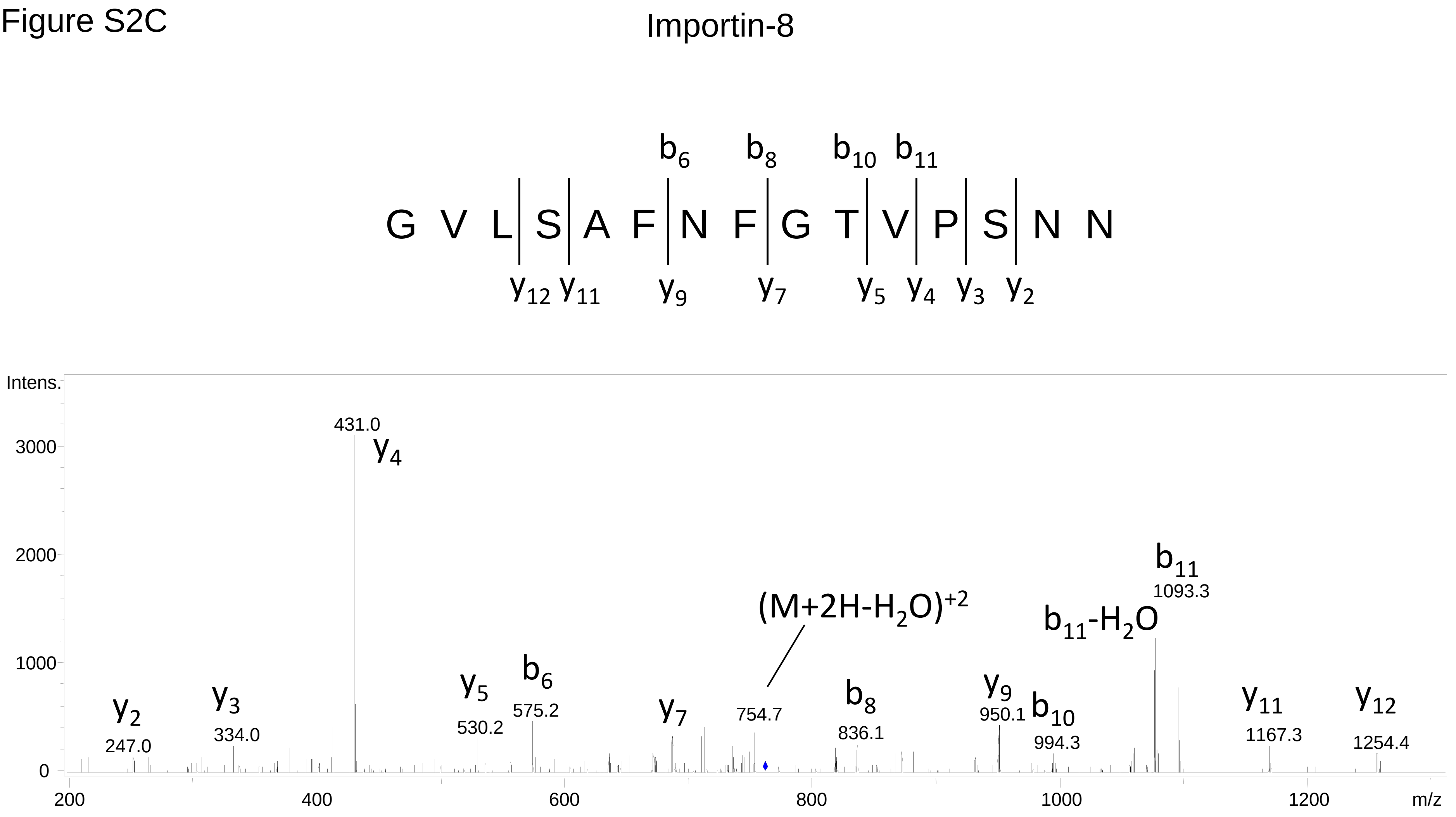
Importin-8
b6 b8 b10 b11
G V L S A F N F G T V P S N N
y12 y11 y9 y7 y5 y4 y3 y2
Intens.
3000
2000
1000
0
431.0
y4
b11
1093.3
(M+2H-H2O)+2
b11-H2O
b6
y5
y3
y2
y7
b8
y9
b10
y11
y12
247.0
334.0
530.2
575.2
754.7
836.1
950.1
994.3
1167.3
1254.4
200
400
600
800
1000
1200
m/z

Figure S2D

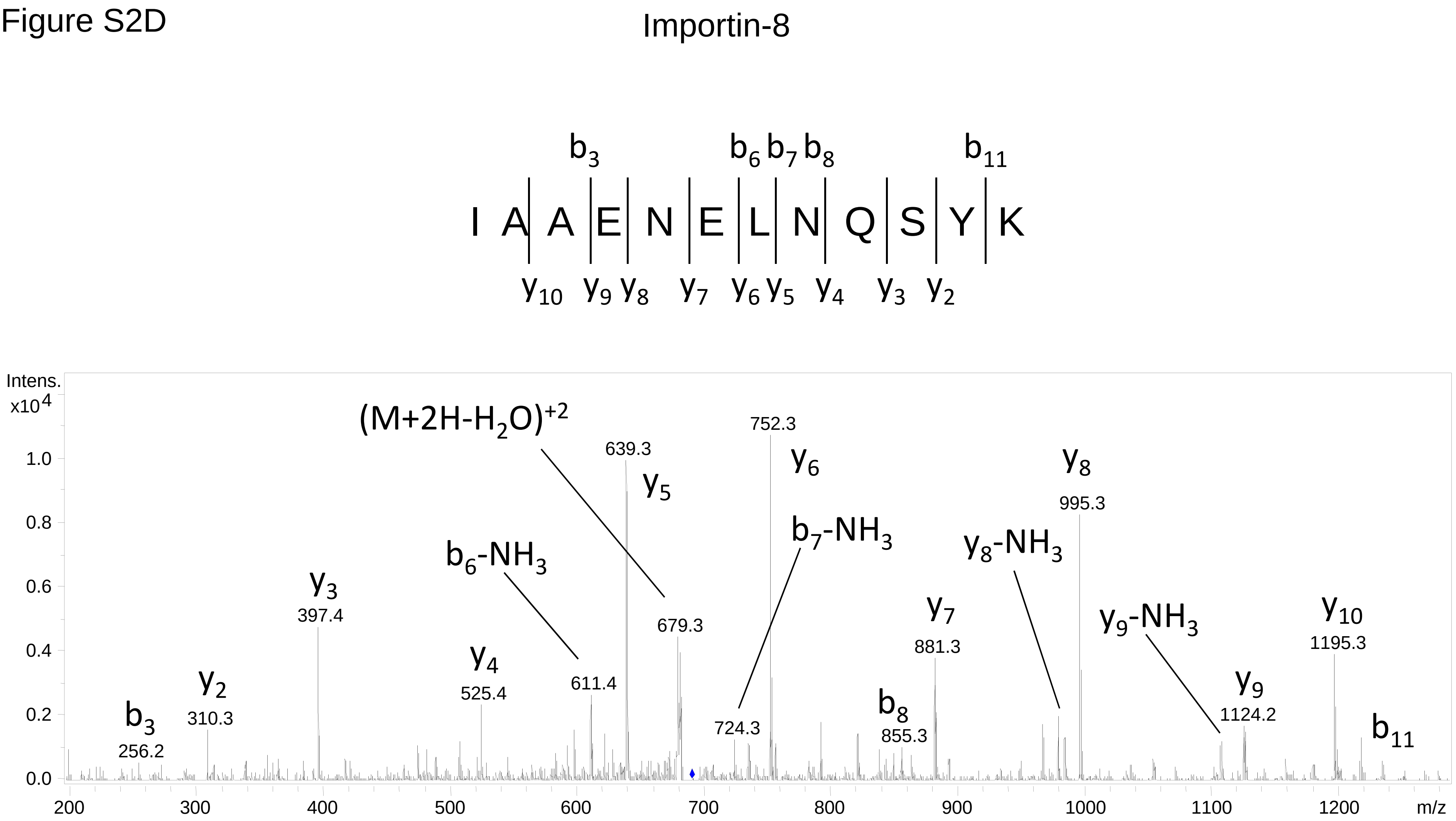

Importin-8
b3 b6 b7 b8 b11
I A A E N E L N Q S Y K
y10 y9 y8 y7 y6 y5 y4 y3 y2
Intens. x10^4
1.0
0.8
0.6
0.4
0.2
0.0
200 300 400 500 600 700 800 900 1000 1100 1200 m/z
(M+2H-H2O)+2
679.3
639.3
y5
752.3
y6
b7-NH3
724.3
b6-NH3
611.4
y3
397.4
y4
525.4
y2
310.3
b3
256.2
y7
881.3
b8
855.3
y8
995.3
y8-NH3
y9-NH3
y9
1124.2
y10
1195.3
b11

Figure S2E

Importin-8

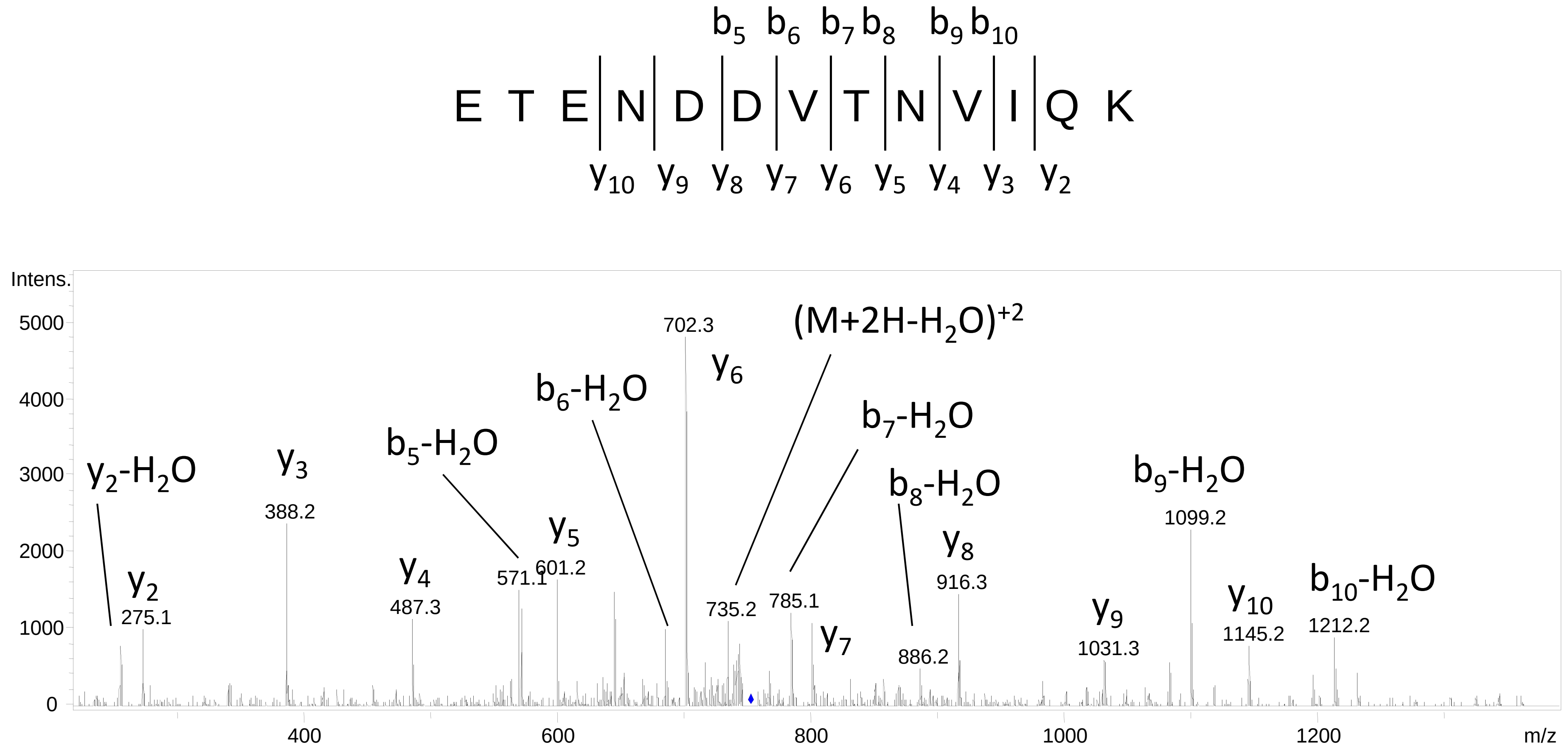

Supplement: Additional file 2: Figure S1. — Identification of importin-5 as a binding partner of hPPIP5K2. Panels a-g show the individual MS/MS spectra from which the data shown in Fig. 3b were obtained. Figure S2. Identification of importin-8 as a binding partner of hPPIP5K2. Panels a-e show the individual MS/MS spectra from which the data shown in Fig. 3c were obtained. [file 12860_2015_63_MOESM2_ESM.pdf]
